# BET Protein Inhibition Regulates Macrophage Chromatin Accessibility and Microbiota-Dependent Colitis

**DOI:** 10.3389/fimmu.2022.856966

**Published:** 2022-03-24

**Authors:** Michelle Hoffner O’Connor, Ana Berglind, Meaghan M. Kennedy Ng, Benjamin P. Keith, Zachary J. Lynch, Matthew R. Schaner, Erin C. Steinbach, Jeremy Herzog, Omar K. Trad, William R. Jeck, Janelle C. Arthur, Jeremy M. Simon, R. Balfour Sartor, Terrence S. Furey, Shehzad Z. Sheikh

**Affiliations:** ^1^ Center for Gastrointestinal Biology and Disease, University of North Carolina at Chapel Hill, Chapel Hill, NC, United States; ^2^ Department of Genetics, Curriculum in Genetics and Molecular Biology, University of North Carolina at Chapel Hill, Chapel Hill, NC, United States; ^3^ Department of Genetics, Curriculum in Bioinformatics and Computational Biology, University of North Carolina at Chapel Hill, Chapel Hill, NC, United States; ^4^ Department of Medicine, Division of Rheumatology, Allergy, and Immunology, University of North Carolina at Chapel Hill, Chapel Hill, NC, United States; ^5^ Department of Pathology, Duke University, Durham, NC, United States; ^6^ Department of Microbiology and Immunology, University of North Carolina at Chapel Hill, Chapel Hill, NC, United States; ^7^ Lineberger Comprehensive Cancer Center, University of North Carolina at Chapel Hill, Chapel Hill, NC, United States; ^8^ UNC Neuroscience Center, University of North Carolina at Chapel Hill, Chapel Hill, NC, United States; ^9^ Carolina Institute for Disabilities, University of North Carolina at Chapel Hill, Chapel Hill, NC, United States; ^10^ Department of Biology, University of North Carolina at Chapel Hill, Chapel Hill, NC, United States

**Keywords:** macrophage, chromatin, inflammation, regulation, Crohn’s disease

## Abstract

**Introduction:**

In colitis, macrophage functionality is altered compared to normal homeostatic conditions. Loss of IL-10 signaling results in an inappropriate chronic inflammatory response to bacterial stimulation. It remains unknown if inhibition of bromodomain and extra-terminal domain (BET) proteins alters usage of DNA regulatory elements responsible for driving inflammatory gene expression. We determined if the BET inhibitor, (+)-JQ1, could suppress inflammatory activation of macrophages in *Il10^-/-^* mice.

**Methods:**

We performed ATAC-seq and RNA-seq on *Il10^-/-^* bone marrow-derived macrophages (BMDMs) cultured in the presence and absence of lipopolysaccharide (LPS) with and without treatment with (+)-JQ1 and evaluated changes in chromatin accessibility and gene expression. Germ-free *Il10^-/-^* mice were treated with (+)-JQ1, colonized with fecal slurries and underwent histological and molecular evaluation 14-days post colonization.

**Results:**

Treatment with (+)-JQ1 suppressed LPS-induced changes in chromatin at distal regulatory elements associated with inflammatory genes, particularly in regions that contain motifs for AP-1 and IRF transcription factors. This resulted in attenuation of inflammatory gene expression. Treatment with (+)-JQ1 *in vivo* resulted in a mild reduction in colitis severity as compared with vehicle-treated mice.

**Conclusion:**

We identified the mechanism of action associated with a new class of compounds that may mitigate aberrant macrophage responses to bacteria in colitis.

## Introduction

Macrophages are key innate immune cells primarily known for their ability to eliminate bacteria, foreign antigens, and debris through phagocytosis, but they also play a central role in the activation of downstream adaptive immune responses (1, 2). Macrophages have specialized roles in maintaining homeostasis in various tissues including the brain, lungs, liver, spleen, peritoneal cavity, and intestines, but these roles can vary (3). Signals from the local microenvironment determine tissue-specific macrophage functional capabilities under homeostatic conditions (4, 5). For example, macrophages found within the intestine are tolerized to the presence of the enteric microbiota, robustly performing phagocytosis when necessary without the release of potent inflammatory cytokines or recruitment of T_H_1 or T_H_17 responses (6, 7). Changes in the local environment or presence of external stimuli are sufficient to prompt rapid activation of macrophages, in which they gain additional functions required to help restore the homeostatic state (4, 5, 8).

DNA regulatory elements (DREs), including enhancers, promoters, and insulators, significantly contribute to the gene expression program and overall function in macrophages (4, 5, 8, 9). Using Chromatin Immuno-Precipitation (ChIP)-seq and Assay for Transposase-Accessible Chromatin (ATAC)-seq to examine the distribution of H3K27ac modifications and nucleosome depleted accessible chromatin, respectively, Lavin et al. determined that tissue resident macrophages have unique enhancer landscapes, resulting in the preference for a distinct set of transcription factors in a tissue-dependent manner (4). Others have demonstrated that the presence of new stimuli resulted in rapid chromatin remodeling to promote expression of relevant response genes (8, 10, 11). In particular, stimulation with lipopolysaccharide (LPS) leads to both phosphorylation of H3S28 and increased acetylation of H3K27, promoting rapid subsequent transcription of inflammatory genes, such as *Il12β*, in bone marrow-derived macrophages (BMDMs) (10, 11). Additionally, Ostuni et al. determined that stimulation-dependent epigenomic memory in BMDMs is established through the formation and usage of latent enhancers, identified by *de novo* H3K4me1 and H3K27ac marks following stimulation (8). Establishment of latent enhancers provides for a more rapid, specific response should macrophages subsequently encounter the same stimulus (8). Together these studies demonstrated that the macrophage epigenetic state, under homeostatic conditions and in response to stimuli, plays a central role in dictating macrophage function. However, epigenetic disturbances that result in dysregulated macrophage functioning are highly understudied.

The inflammatory bowel diseases (IBDs), Crohn’s disease (CD) and ulcerative colitis, are chronic inflammatory conditions of the intestine driven by exaggerated immune responses toward enteric bacteria in a genetically susceptible host (12). Macrophage loss of tolerance toward bacteria is a key event attributed to the pathogenesis of CD (13–24). This is primarily driven by increased production of *Il12β*/IL-12p40, a subunit in the heterodimeric proteins, IL-12 and IL-23, which are responsible for downstream activation of T_H_1 immune responses (13, 17, 23). The *Il10^-/-^* mouse is a genotype-driven, microbiota-dependent mouse model in which loss of production of IL-10 by T regulatory cells results in inflammatory activation of macrophages leading to a classical T_H_1 inflammatory immune response (25–28). This dysregulated inflammatory response results in major pathological changes, including increased immune infiltrate, epithelial hypoplasia, crypt abscesses, and focal ulcers throughout the caecum, colon and rectum and leaves mice vulnerable to death resulting from bacterial-induced septic shock (25). Previously, we identified altered chromatin remodeling in macrophages from both *Il10^-/-^* mice and colonic tissue isolated from CD patients (9). Using BMDMs and lamina propria macrophages (LP MΦs) isolated from wild-type (WT) and *Il10^-/-^* mice, we determined that both macrophage subsets had genome-wide chromatin accessibility changes associated with bacterial stimulation as well as genotype-specific modifications associated with the loss of IL-10. Increased accessibility was most frequently associated with LPS stimulation in BMDMs while increased accessibility in LP MΦs was most frequently associated with the loss of IL-10. Interestingly, the accessible chromatin regions associated with these two categories corresponded to a common set of genes, particularly those involved with the inflammatory response, including *Nos2*, *Irf1*, *Il7r*, and *Rel.* These data emphasize the importance of genetic predisposition as a key driver of chromatin structure that is independent of bacteria in addition to unique chromatin responses driven by bacterial stimuli. A major challenge is how to prevent the formation of an aberrant chromatin state in macrophages that promotes chronic intestinal inflammation.

The bromodomain and extra-terminal domain (BET) protein family consists of four distinct proteins known for their ability to recognize acetylated lysine residues on histone proteins, which contributes to gene regulatory activity (29). Small molecule binding of the BET proteins’ two bromodomain active sites prevents the recognition of acetylated lysines resulting in attenuated inflammatory responses (30, 31). Nicodeme et al. originally demonstrated BET protein binding increased upon LPS stimulation of BMDMs, but the addition of the small molecule, I-BET151, was sufficient to decrease BET protein binding and decrease expression of LPS responsive genes (30). Studies using other BET inhibitors, such as (+)-JQ1, confirmed that inhibition of BET proteins attenuates LPS- and/or IFN-γ-induced secretion of these cytokines in BMDMs and *in vivo* murine models of endotoxic shock (31–34). Although BET proteins have been implicated in direct remodeling of chromatin, previous studies have not examined the full of extent of their role shaping chromatin accessibility in the LPS response (35, 36). Additionally, findings from Cheung et al. suggest that BET inhibitors are capable of limiting chronic inflammatory conditions, such as adoptive T-cell transfer-induced colitis by limiting expansion of T_H_1 and T_H_17 cell differentiation (37). However, it is unclear if the *in vitro* responses observed in macrophages, activated upstream of T cells *in vivo*, can be recapitulated and what is the mechanism by which BET inhibitors function to limit inflammatory gene expression.

In this study, we sought to determine if inhibition of BET proteins using the inhibitor (+)-JQ1 is sufficient to mitigate chromatin remodeling associated with the inflammatory state in macrophages when challenged with LPS. Our findings indicate that treatment of macrophages with (+)-JQ1 prior to LPS stimulation limited genome-wide changes in chromatin accessibility, particularly at regions distal to transcription start sites. Concomitant changes in gene expression were also observed, where combinations of treatment with (+)-JQ1 and LPS stimulation revealed 10 distinct classes of genes based on their patterns of expression. Expression of approximately 90% of LPS-induced genes identified was attenuated with (+)-JQ1 treatment. Analysis of differentially accessible chromatin regions nearby but distal to transcription start sites (TSSs) of LPS-induced genes revealed enrichment for AP-1 and IRF transcription factor binding motifs and that (+)-JQ1 treatment prevented remodeling at approximately 1,100 AP-1 and IRF motif target sequences. Finally, to evaluate efficacy of (+)-JQ1 *in vivo*, we treated *Il10^-/-^* with (+)-JQ1 following colonization with fecal slurries and determined (+)-JQ1 results in a mild reduction in colitis severity. Together, these data highlight that BET protein inhibition by (+)-JQ1 is sufficient to reduce LPS-induced chromatin remodeling, giving rise to attenuated inflammatory responses in *Il10^-/-^* macrophages more consistent with the function of LP MΦs.

## Materials and Methods

### Mice

Wild-type (WT) and *Il10^-/-^* mice on the C57BL/6J background maintained under specific pathogen free (SPF) conditions were used to generate BMDMs. Germ free *Il10^-/-^* mice (C57BL/6J background) were bred and maintained by the National Gnotobiotic Rodent Resource Center (NGRRC) and used to evaluate the clinical efficacy of (+)-JQ1.

### Colonization and Treatment of Mice

8-12-week-old *Il10^-/-^* mice (C57BL/6J background; 22 male mice and 10 female mice) were removed from germ free housing and placed in isolation in a BSL2 facility for the duration of these experiments. Treatment group assignments were balanced for gender. Mice were injected with 50 mg/kg (+)-JQ1 (MedChemExpress, #HY-13030) resuspended in 20% sulfobutylether-β-cyclodextrin (SBE-β-CD) (MedChemExpress, #HY-17031) or 10% DMSO diluted in 20% SBE-β-CD. Mice were subsequently colonized with slurries made from feces of C57BL/6J mice raised under SPF conditions resuspended in pre-reduced anaerobic 1x PBS by oral and rectal swabbing. Additional injections were given on Days 3, 6, 9, and 12. Weight was measured daily through to harvest on Day 14.

### Macrophage Isolation and Stimulation

BMDMs from 12-week-old male (3 *Il10^-/-^* and 4 WT) mice were harvested and cultured as previously described in biological triplicate (9). BMDMs were counted and re-plated in duplicate for RNA- and ATAC-seq experiments. Macrophages were treated with 500nM (+)-JQ1 (MedChemExpress #HY-13030), 10 ng/mL recombinant IL-10 (PeproTech, #210-10), or vehicle control for 12 hours followed by the addition of 50 ng/mL LPS (InvivoGen, #tlrl-peklps) for 4 hours. Untreated, unstimulated WT or *Il10^-/-^* BMDMs served as an additional control and remained in culture for 16 hours. Samples were collected in TRIzol or freezing media for RNA- and ATAC-seq experiments, respectively. Cell culture supernatants were collected and stored for cytokine secretion analyses using Luminex.

### RNA-Seq

RNA was isolated from murine BMDMs using the Norgen Biotek Corp. Total RNA Purification Kit (Cat. 17200) according to the manufacturer’s protocols. These kits use column-based DNase treatment to eliminate DNA contamination.

RNA-seq libraries were prepared using the Illumina KAPA Stranded RNA-seq Kit with RiboErase (HMR). Paired-end (50bp) sequencing was performed on the Illumina HiSeq 4000 platform (Gene Expression Omnibus [GEO] accession no. 184563). Reads were extracted using TagDust v1.13 and aligned to the mm9 genome assemblies using STAR with default parameters (9, 38, 39). Reads were quantified using RSEM v1.2.31 with default parameters (9, 40). PCA was performed using the prcomp function in R on DESeq2 normalized variance stabilizing transformation (VST) transformed counts for the top 1,000 most variably expressed genes (41).

Pairwise differential gene expression was determined using the DESeq2 Wald test (41). Pairwise DEGs between LPS stimulated and unstimulated controls, between LPS stimulated + (+)-JQ1 treated and unstimulated controls, and LPS stimulated and LPS stimulated + (+)-JQ1 treated were determined, the latter used to indicate whether (+)-JQ1 treatment promoted increased or decreased relative gene expression. To focus on genes significantly affected by (+)-JQ1 treatment during LPS stimulation, gene expression for individual biological replicates for LPS stimulated and LPS + (+)-JQ1 conditions were normalized to the average expression across unstimulated *ll10^-/-^* BMDMs and DEGs (base mean expression ≥ 10 and FDR < 0.05) were identified using the likelihood ratio test (LRT) in DESeq2 (41). Distinct gene expression classes (I – X) were identified based on varying combinations of levels of statistical significance and log_2_ fold-change of individual Wald tests (**Supplementary Table 1**). Z-scores were calculated based on the log_2_ fold-change of individual biological replicates for LPS stimulated and LPS stimulated + (+)-JQ1 treated samples normalized to the average of the unstimulated and untreated *Il10^-/-^* BMDMs. These z-scores were used to generate heatmaps of the ten classes. Enriched KEGG pathways (FDR < 0.05) were identified using Enrichr (42).

### ATAC-Seq

ATAC-seq was performed as previously described (43). To prepare nuclei, cells were pelleted and washed with cold PBS followed by lysis using cold lysis buffer (10mM Tris-HCl, 10mM NaCl, 3 mM MgCl2, and 0.1% NP-40). Pelleted nuclei underwent transposition using the Nextera DNA Library Prep Kit (Illumina #FC-121-1030). Samples were resuspended in the transposition reaction (12.5µL 2x TD buffer, 2 µL transposase, and 10.5 µL nuclease-free water) and incubated at 37C for 1 hour at 1000 RPM. Transposed DNA samples were purified using the Qiagen MinElute Kit (#28204) followed by amplification using 1x PCR master mix (NEB #M0541S) and 25µM Ad1_noMX and Ad2.* indexing primer for 10-14 cycles. Libraries were purified and size selected by magnetic separation using Agencourt AMPure XP magnetic beads (Beckman Coulter #A63880). Paired-end (50bp) sequencing was performed on the Illumina HiSeq 4000 platform (GEO accession no. 183564). Data was processed using PEPATAC v0.9.0 with default parameters (44). Uniquely mapped reads were aligned to the mm9 genome using bowtie2 (45). Peaks were called on individual samples using MACS2 (46, 47). ChIPSeeker was used to classify peaks as promoter or distal, where any peak that that did not fall in the -1000bp/+100bp from the TSS of mm9 reference genes was classified as distal (48). BEDTools was used to determine distal peaks +/-25kb from the TSS (50kb window) of each gene (49).

PCA was performed using the prcomp function in R on DESeq2 normalized, variance stabilizing transformation (VST) transformed counts for the top 10,000 most variably accessible peaks (41). Promoter and distal peaks were calculated and scaled with the computeMatrix tool from DeepTools v 3.5.0 and plotted using either plotHeatmap or plotProfile (50). Differentially accessible regions (DARs) across the union set of peaks was determined using DESeq2 (41). DARs with a |log_2_ fold-change| > 1 were identified using VennDiagram v1.6.20 (51). VST transformed counts were used for Spearman-ranked correlation analyses.

For each gene, DARs +/-25kb of the TSS were associated with the gene. Enrichments of DARs around genes in expression-defined categories (I – X) were calculated using a hypergeometric test. Known transcription factor motif analysis was performed with *findMotifsGenome* from HOMER using the middle 500bp window of DARs (FDR < 0.05). The *annotatePeaks* command was used to identify the location of enriched transcription factor motifs (FDR < 0.10) (52, 53). Motif matrices were clustered using Regulatory Sequence Analysis Tools (RSAT) using the Ncor metric for motif-to-motif similarity matrix with the lower thresholds set to 5 for width, 0.7 for Pearson correlation, and 0.4 for relative width-normalized Pearson correlation (54).

### Luminex

Cytokine levels for BAFF, CRP, IL-12p70, IL-6, IL-27, LDLR, MCP-1, MCP-2, MIP-1α, and TNF-α found in cell culture medium following WT and *Il10^-/-^* BMDM stimulation were measured by Luminex (R&D) and analyzed on a Bio-plex 200 (Bio-Rad Laboratories). Cytokine levels were calculated based on the standard curve for each analyte. Samples were plated in technical duplicate and biological triplicate. Data-points are representative of the average of technical duplicates for each sample.

### Histology

Slides containing sections of proximal and distal colon were prepared for H & E staining. Two independent scorers blinded to the experimental and control groups performed histological analysis using an established scoring system for evaluating goblet cell loss and submucosal infiltrate in animal models (55). Histological sub-scores were added together to generate a composite histology score.

### RNA Extraction and qPCR

Total RNA was isolated from WT and *Il10^-/-^* BMDMs for all experimental conditions and caecal tissue taken from GF (Day 0) and colonized (Day 14) *Il10^-/-^* mice using the Norgen Biotek Corp. Total RNA Purification Kit (Cat. 17200). cDNA was derived from 500ng RNA by reverse transcriptase using the High Capacity cDNA Reverse Transcription Kit (Applied Biosystems, 4368814) according to the manufacturer’s specifications. Quantitative real-time PCR was performed using 10ng cDNA with the PowerUp SYBR Green Master Mix (Applied Biosystems, A25741) and 10 µM of forward and reverse primers for *Il12β* (F: 5’ CGCAAGAAAGAAAAGATGAAGGAG 3’ R: 5’ TTGCATTGGACTTCGGTAGATG 3’), *Tnf* (F: 5’ ACCCTCACACTCAGATCATCTTCTC 3’ R: 5’ TGAGATCCATGCCGTTGG 3’) and *β-actin* (F: 5’ AGCCATGTACGTAGCCATCCAG 3’ R: 5’ TGGCGTGAGGGAGAGCATAG 3’). Reactions were performed in triplicate for each biological sample. Fold-change was calculated by determining ΔΔCt for all reactions followed by normalization to the average ΔΔCt value for unstimulated BMDMs and GF animals.

### Flow Cytometry

*Il10^-/-^* BMDMs were cultured for 16 hours with 500nM (+)-JQ1. Cells were washed with cold 1x PBS followed by staining with LIVE/DEAD Fixable Blue Dead Cell Stain Kit (1:1000; Invitrogen L23105). Cell cycle changes in (+)-JQ1 treated *Il10^-/-^* BMDMs were assessed using the BD Pharmigen BrdU Flow kit according to the manufacturer (559619). Briefly, *Il10^-/-^* BMDMs were co-cultured with BrdU and treated with (+)-JQ1 for 16 hours followed by fixation and permeabilization. Cells were treated with DNase followed by staining with FITC-conjugated anti-BrdU.

Bulk LPMCs from *Il10^-/-^* mice were washed with cold 1x PBS and stained for viability followed by cell-surface marker staining for CD45 (1:100; Clone 30-F11, BioLegend), CD3ε (1:300; Clone 145-2C11, BioLegend), CD19 (1:200; Clone 6D5, BioLegend), CD11b (1:200; M1/70, BD Biosciences), CD11c (1:200; Clone N418, BioLegend), and F4/80 (1:200; Clone BM8, BioLegend) diluted in staining buffer (5% FBS/PBS). All samples were fixed with 4% PFA. Data was acquired with the FACSDIVA software using the BD LSR II and analyzed using FlowJo version 10.7.1.

### Statistics

Differential analyses of ATAC-seq and RNA-seq data were performed using DESeq2 with FDR adjusted *P* values used to measure statistical significance (41). Spearman rank correlations of VST transformed counts were calculated using RStudio Version 1.2.5033. Known motif enrichment was determined using HOMER, which generates *P* values by screening a library of reliable motifs against target and background sequences. GraphPad Prism 8 for Mac (Graphpad Software Inc.) was used for all other statistical analyses. Statistical significance of change in the number of DARs with a |log_2_ fold-change| > 0.5 for each condition was determined using a 2x2 chi-square test. Statistical significance comparing the absolute log_2_ fold-change values for LPS stimulated and LPS stimulated + (+)-JQ1 treated samples were determined by Wilcoxon match-paired signed rank tests. Statistical differences in relative gene expression and cytokine secretion for the LPS and LPS + (+)-JQ1 conditions were determined using paired, parametric t-tests. Statistical differences in relative gene expression and cytokine secretion for the LPS, LPS + (+)-JQ1 and LPS + IL-10 conditions were determined using one-way ANOVA followed by *post hoc* analyses using Tukey’s multiple comparisons test. Paired parametric t-tests were used to determine statistical significance for (+)-JQ1 and DMSO treated BMDMs for the evaluation of the number of cells in S-phase and percentage of viable cells. All other statistics were determined using a 2-tailed unpaired, nonparametric, student’s t test. For all tests, except for motif analysis, *P*_adj_ < 0.05, empirical *P* < 0.05, or *P* < 0.05 were considered statistically significant. For motif analysis, *P*_adj_ < 0.1 was considered statistically significant.

### Study Approval

All animal experiments were performed in accordance with protocols approved by the Institutional Animal Care and Use Committee of the University of North Carolina at Chapel Hill (19-108.0).

## Results

### Inhibition of BET Proteins Limits LPS-Induced Changes in Macrophage Chromatin Accessibility Genome-Wide

**Figure 1:**
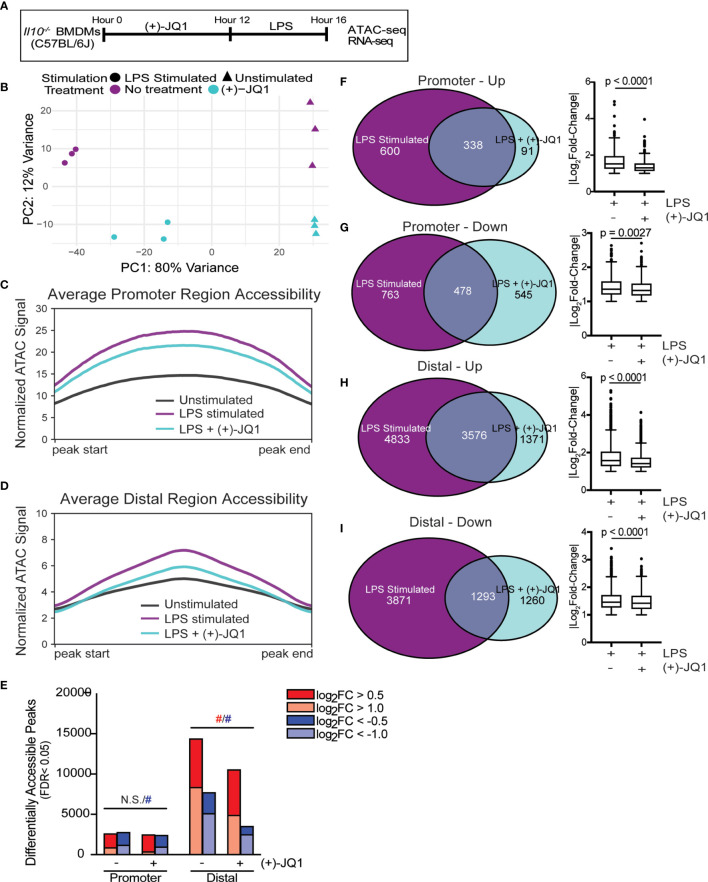
(+)-JQ1 treatment prevents LPS-induced chromatin accessibility changes in distal regions. **(A)** Schematic of experimental workflow. **(B)** Principle component analysis (PCA) of chromatin accessibility profiles for samples that remained unstimulated (triangles) or were stimulated with LPS (circles) in the absence (purple) or presence (aqua) of (+)-JQ1. Average chromatin accessibility signal profiles for **(C)** promoter regions (-1000/+100 bases) and **(D)** distal regions for unstimulated (black), LPS stimulated (purple) and LPS + (+)-JQ1 (aqua) samples. **(E)** Identification of regions that differentially increase (red) or decrease (blue) in accessibility during LPS stimulation with or without (+)-JQ1 as compared with unstimulated BMDMs at promoter (left) and distal (right) regions (FDR < 0.05). Red and blue hashtags indicate difference in the number of regions with increased (red) or decreased (blue) accessibility with (+)-JQ1 is statistically significant using a chi-square test. Identification of peaks shared with |log_2_ fold-change >1| (left) and comparison of average log_2_ fold-change values using Wilcoxon sum rank tests for **(F)** promoters increasing in accessibility, **(G)** promoters decreasing in accessibility, **(H)** distal regions increasing in accessibility, and **(I)** distal regions decreasing in accessibility. FDR adjusted P values determined using DESeq2. N = 3 biological replicates. N.S. stands for Not Significant.

Genome-wide epigenetic changes, including post-translational histone modifications and chromatin accessibility, have been heavily annotated in macrophages upon LPS stimulation (8–10, 56). Increased BET protein binding has been associated with expression of LPS-induced inflammatory genes (30–33, 37). To comprehensively determine the role of BET proteins in LPS-induced chromatin remodeling, we treated *Il10^-/-^* BMDMs with (+)-JQ1 for 12 hours prior to challenge with LPS for 4 hours and evaluated changes in chromatin accessibility by ATAC-seq (**Figure 1A**). Treatment with (+)-JQ1 did not affect cell viability (**Supplementary Figure 1A**) or cell cycle progression (**Supplementary Figure 1B**).

Principle component analysis (PCA) of chromatin accessibility profiles in *Il10^-/-^* BMDMs (**Figure 1B**) showed the first principle component (PC1) stratified samples based on the presence or absence of LPS stimulation, demonstrating that LPS is a primary driver of chromatin accessibility changes. Samples were further segregated along the second PC based on treatment with (+)-JQ1. We note that samples treated with (+)-JQ1 prior to LPS stimulation clustered more closely to unstimulated samples along PC1 than untreated LPS stimulated samples (**Figure 1B**), suggesting (+)-JQ1 mitigates some LPS-induced chromatin accessibility changes in *Il10^-/-^* BMDMs.

Both LPS stimulation and the loss of IL-10 expression were previously shown to affect genome-wide chromatin accessibility in *Il10^-/-^* BMDMs (9). To determine how (+)-JQ1 modifies chromatin accessibility during LPS stimulation at both promoter (n = 36,670) and distal (n=157,614) sites genome-wide, we plotted average chromatin accessibility (**Figures 1C, D**) and quantified the overall number and magnitude of differentially accessible regions (DARs; FDR < 0.05) for both LPS and LPS + (+)-JQ1 conditions as compared with unstimulated *Il10^-/-^* BMDMs (**Figure 1E**). Average chromatin accessibility at both promoter and distal sites decreased with (+)-JQ1 treatment as compared with LPS stimulation alone (**Figures 1C, D**). However, the relative decrease in accessibility was greater at distal sites (**Figure 1D**) as compared with promoter sites (**Figure 1C**). The number of promoter DARS that had increased accessibility upon LPS stimulation was roughly equivalent under each condition with no statistically significant differences (**Figure 1E**). In contrast, (+)-JQ1 treatment prior to LPS stimulation resulted in fewer promoter DARs with decreased accessibility as compared with LPS stimulation alone (**Figure 1E**; p=8.54x10^-8^). In contrast at distal sites, both the overall number of DARs as well as the percentage of high magnitude DARs were decreased substantially with (+)-JQ1 treatment (**Figure 1E**; p<2.2x10^-16^ for both increased and decreased). Focusing on DARs with high magnitude changes at both promoter and distal sites, we found greater concordance across the two conditions when accessibility was increased by LPS stimulation than when decreased (**Figures 1F–I**). Even amongst these DARs, though, (+)-JQ1 treatment significantly decreased the average magnitude (|log_2_FC|) for all four categories (**Figures 1F–I**, right; Wilcoxon sum rank test, p < 0.005). Based on these data, we conclude that (+)-JQ1 treatment overall attenuates the effect of LPS on changes to genome-wide chromatin accessibility and has a greater impact on DARs at distal sites.

### Exogenous IL-10 Treatment Drives Changes in Chromatin Accessibility Distinct From BET Inhibition

We previously showed that supplementation with exogenous IL-10 had minimal effects on genome-wide chromatin remodeling in *Il10^-/-^* BMDMs (9). To determine how IL-10 treatment compared to (+)-JQ1 in limiting LPS-induced chromatin remodeling, we treated *Il10^-/-^* BMDMs with IL-10 for 12 hours followed by LPS challenge for 4 hours. PCA revealed that samples treated with IL-10 clustered separately from samples treated with (+)-JQ1 and from untreated samples, regardless of LPS stimulation (**Supplementary Figure 2A**). IL-10 treated samples had decreased promoter accessibility compared with (+)-JQ1 treated samples, on average (**Supplementary Figure 2B**). In contrast, (+)-JQ1 treated samples had decreased accessibility at distal regions compared to IL-10 samples (**Supplementary Figure 2C**). When comparing with unstimulated and untreated *Il10^-/-^* BMDMs, IL-10 treatment prior to LPS stimulation resulted less promoter DARs as compared with LPS stimulation alone or with (+)-JQ1 treatment (**Supplementary Figure 2D**; p=2.003x10^-15^ for increased, p<2.2x10^-16^ for decreased). In contrast, the overall number of DARs (increased and decreased together) detected at distal sites with IL-10 treatment prior to LPS stimulation were similar to what was observed with LPS stimulation alone. When separately testing DARs with increased or decreased accessibility, differences in each were statistically different by chi-square analysis (**Supplementary Figure 2D**; 1.811x10^-15^ for increased, 2.2x10^-16^ for decreased). To better quantify effects of treatments on chromatin accessibility changes with LPS stimulation, we correlated normalized signals across all accessible regions between each of LPS stimulated alone, LPS + (+)-JQ1, and LPS + IL-10 with unstimulated *Il10^-/-^* BMDMs (**Supplementary Figures 2E–G**). We found (+)-JQ1 treated samples were the most correlated with unstimulated samples suggesting a greater impact than IL-10 on limiting chromatin remodeling. Together, these data show (+)-JQ1 better preserves a non-inflammatory chromatin state and that IL-10 signaling affects chromatin remodeling in a distinct manner.

### BET Protein Inhibition Attenuates LPS-Induced Expression of Inflammatory Genes

**Figure 2:**
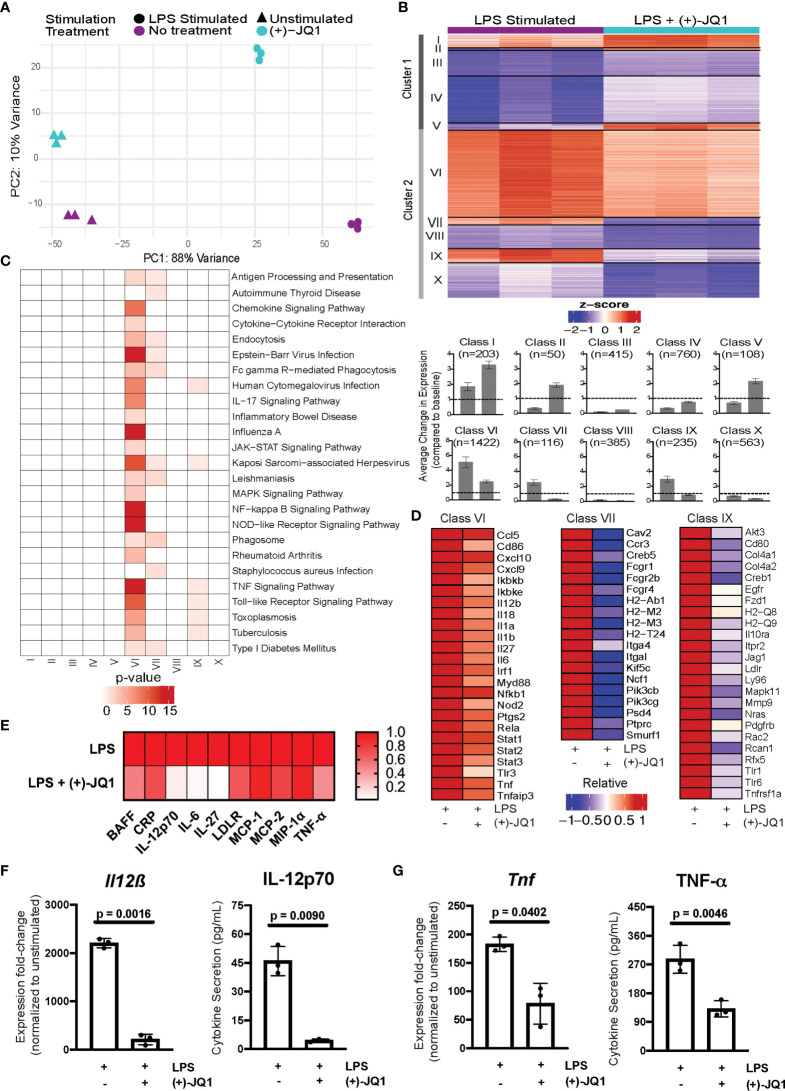
(+)-JQ1 treatment attenuates LPS-induced inflammatory gene expression in *Il10^-/-^* macrophages. **(A)** Principle component analysis (PCA) of gene expression profiles for samples that remained unstimulated (triangles) or were stimulated with LPS (circles) in the absence (purple) or presence (aqua) of (+)-JQ1. **(B)** Representative heatmap of calculated z-scores based on log_2_ fold-change of individual replicates for the LPS and LPS + (+)-JQ1 conditions normalized to the average of unstimulated, untreated *Il10^-/-^* BMDMs. Genes were identified and divided into 2 major clusters and sub-divided into 5 classes through differential analyses outlined in **Supplementary Table 1**. Bar graphs represent average change in expression for LPS and LPS (+)-JQ1 conditions normalized to baseline. **(C)** KEGG pathway analysis for the genes identified in the 10 classes in **(B)**. **(D)** Heatmaps of representative genes from Classes VI, VII, and IX that correspond to genes induced by LPS that are attenuated with (+)-JQ1. **(E)** Heatmaps of representative inflammatory cytokines induced by LPS that are attenuated with (+)-JQ1. Gene expression (qRT-PCR; left panels) and cytokine secretion (Luminex; right panels) analyses for **(F)** *Il12β* and IL-12p70, and **(G)** *Tnf* and TNF-α. Significance determined using paired parametric, student’s t tests. N = 3 biological replicates.

LPS stimulation of *Il10^-/-^* BMDMs results in robust expression of key inflammatory genes, such as *Il12β*, that is greater than expression observed in WT BMDMs (20, 22). To better understand how BET protein inhibition affects gene expression in response to LPS stimulation, we performed RNA-seq using matched BMDM samples (**Figure 1A**). Similar to our initial ATAC-seq analyses, PCA on these data revealed the primary source of variation is due to LPS stimulation (PC1; **Figure 2A**) while (+)-JQ1 treatment orthogonally contributes to variation (PC2). Again, samples treated with (+)-JQ1 prior to LPS stimulation clustered closer to unstimulated samples along PC1 than untreated LPS stimulated samples.

To determine which genes were affected by LPS stimulation both with and without (+)-JQ1 treatment, we performed pairwise comparisons with unstimulated samples separately for each. We identified a total of 10,656 differentially expressed genes (DEGs) present in at least one of these comparisons. We then used a likelihood ratio test (LRT) to determine more specifically which genes were significantly affected by (+)-JQ1 during LPS-stimulation, resulting in 4,257 genes.

DEGs were clustered based on the direction of effect of (+)-JQ1 treatment compared with no treatment. Cluster 1 (n = 1,536 genes) contained genes that had higher expression after LPS stimulation when treated with (+)-JQ1 compared with LPS stimulation alone, while Cluster 2 (n = 2,721 genes) contained those with lower relative expression (**Figure 2B**). Next, within each cluster we identified five distinct classes based on statistical significance and log_2_FC magnitude associated with the pair-wise differential expression analyses with unstimulated samples (**Supplementary Table 1**). In Cluster 1, three small classes contained genes that were significantly up-regulated by LPS with (+)-JQ1 treatment (log_2_FC > 1) but were either weakly induced by (Class I; n = 203 genes), down-regulated by (Class II; n = 50 genes), or showed minimal response to (Class V; n = 108 genes) LPS stimulation alone. In the other two larger classes (Class III, n = 415 genes; Class IV, n = 760 genes), (+)-JQ1 treatment also resulted in relatively higher expression compared to no treatment, but overall expression levels of these genes regardless of treatment were below baseline expression in unstimulated, untreated macrophages (**Figure 2**). Pathway enrichment analysis of all genes in Cluster 1 revealed a slight enrichment for Longevity Regulating, Mismatch Repair, and Systemic Lupus Erythematosus pathways, but no additional pathways (**Supplementary Figure 3A**).

In Cluster 2, which consists of genes whose relative expression is lower when treated with (+)-JQ1 during LPS stimulation (**Figure 2B**), we find nearly all (~90%) genes significantly induced during LPS stimulation alone (log_2_FC > 1). The most robustly up-regulated of these genes were grouped into the largest Class VI (n = 1,422 genes), which contained genes upregulated by LPS stimulation regardless of (+)-JQ1 treatment, but where pre-treating with (+)-JQ1 resulted in a 2-fold decrease in expression compared with LPS stimulation alone on average. Pathway analysis using these genes showed enrichment for more than 20 pathways associated with inflammatory and viral response (**Figure 2C**) and included many pro-inflammatory genes such as *Il12β*, *Il6*, and *Tnf* (**Figure 2D**). Luminex analyses were used to determine if (+)-JQ1-mediated attenuation could also be observed at the protein level. Quantification of seven cytokines found in Class VI (IL-12p70, IL-27, IL-6, MCP-1, MCP-2, MIP-1α, and TNF- α) confirmed that (+)-JQ1 pre-treatment resulted decreased secretion relative to LPS stimulated cells alone (**Figure 2E**). Statistical analyses confirmed that expression of *Il12β*/IL-12p70 (**Figure 2F**) and *Tnf*/TNF-α (**Figure 2G**) at the mRNA (qRT-PCR, left) and protein levels (Luminex, right) was statistically significantly reduced with (+)-JQ1 pre-treatment as compared with LPS stimulation alone.

Class VII (n = 116 genes) and Class IX (n = 235 genes) consisted of genes induced by LPS stimulation alone, albeit less robustly than those in Class VI, but whose expression levels when treated with (+)-JQ1 treatment were lower than or similar to expression in unstimulated *Il10^-/-^* BMDMs, respectively (**Figure 2B**). Class VII genes were enriched for macrophage functions, such as antigen presentation and phagocytosis, while Class IX genes were involved in bacterial and viral response pathways as well as TNF signaling (**Figure 2C**). The remaining two classes (Class VIII, n = 385 genes; Class X, n = 563 genes) were down-regulated upon LPS stimulation regardless of (+)-JQ1 treatment, but whose expression was reduced to a greater extent when treated with (+)-JQ1 (**Figure 2B**). Neither class showed enrichment for immune pathways (**Figure 2C**).

### Exogenous IL-10 Exerts Limited Anti-Inflammatory Effects on LPS-Induced Gene Expression

IL-10 negatively regulates expression of *Il12β* and other inflammatory genes (20, 22). We wanted to better understand how exogenous IL-10 affects the *Il10^-/-^* BMDM response to LPS and to compare this with (+)-JQ1 treatment. PCA revealed that IL-10 treatment prior to LPS stimulation resulted in transcriptional profiles markedly similar to untreated LPS stimulated *Il10^-/-^* BMDMs (**Supplementary Figure 3B**), including across genes in our ten previously defined LPS response classes (**Supplementary Figure 3C**). Expression fold-changes upon LPS stimulation of IL-10 treated samples varied across key inflammatory genes found in Class VI. Some genes, such as *Il12β*, did have attenuated responses comparable to (+)-JQ1 treated samples, but most other genes, such as *Nod2*, showed increased expression compared with (+)-JQ1 treatment (**Supplementary Figure 3D**). Similar changes associated with IL-10 were also observed at the protein level by Luminex (**Supplementary Figure 3E**). Quantitative analyses using qRT-PCR (left) and Luminex (right) for *Il12β*/IL-12p70 (**Supplementary Figure 3F**) and *Tnf*/TNF-α (**Supplementary Figure 3G**) confirmed that IL-10 pre-treatment significantly reduced LPS-induced expression, although (+)-JQ1 was slightly more effective in attenuating *Il12β*/IL-12p70. In contrast, IL-10 treatment had little effect on responses of Class VII and IX genes to LPS (**Supplementary Figure 3D**). Collectively, these results demonstrate that (+)-JQ1 treatment has a strong effect on genes induced by LPS stimulation, which were more extensive than with IL-10 treatment and resulted in down-regulation of genes implicated in the inflammatory response.

### Effects of (+)-JQ1 on WT LPS-Stimulated Cells Has Less Pronounced Effects

To determine the effects of (+)-JQ1 pre-treatment on WT LPS-stimulated BMDMs, WT cells were cultured and stimulated in the exact same manner as *Il10^-/-^* BMDMs (**Figure 1A**) followed by qRT-PCR and luminex analyses to determine changes for select inflammatory genes. Like *Il10^-/-^* BMDMs, treatment of WT BMDMs with (+)-JQ1 did not affect cell viability (**Supplementary Figure 4A**) or cell cycle progression (**Supplementary Figure 4B**). Overall, WT BMDMs responded less robustly to LPS stimulation as demonstrated by the variation in the individual datapoints for *Il12β*/IL-12p70 (**Supplementary Figure 4C**) and *Tnf*/TNF-α (**Supplementary Figure 4D**). While (+)-JQ1 pre-treatment decreased expression at the gene expression and cytokine secretion level for both genes, these findings were not statistically significant (**Supplementary Figures 4C, D**). While IL-10 pre-treatment had a stronger effect at the transcriptional level as compared with cytokine secretion, none of these findings were statistically significant (**Supplementary Figures 4C, D**). Together, these data suggest that (+)-JQ1 and IL-10 exert anti-inflammatory effects on WT BMDMs, but the overall effects are less pronounced as compared with our findings in *Il10^-/-^* BMDMs.

### (+)-JQ1 Restricts Chromatin Accessibility at Distal Regulatory Elements of Class VI Genes

**Figure 3:**
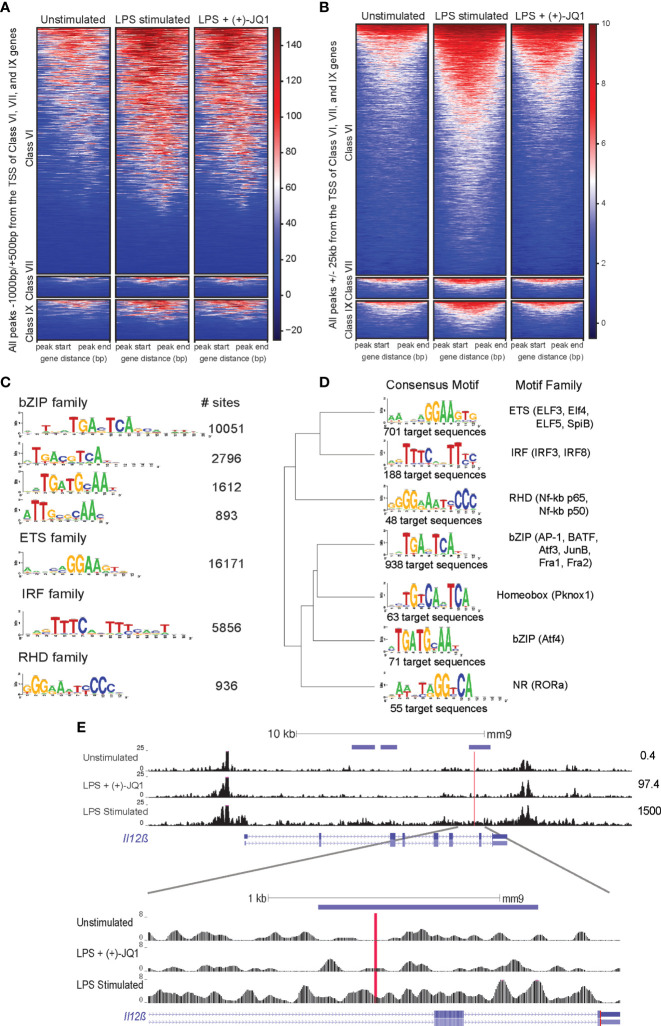
(+)-JQ1 prevents LPS-induced chromatin remodeling at putative AP-1 and IRF regulatory elements associated with class VI genes. Heatmaps of accessible chromatin during unstimulated (left), LPS stimulated (center) or LPS + (+)-JQ1 (right) conditions for Classes VI (top), VII (center), and IX (bottom) for ChIPSeeker identified **(A)** promoter regions and **(B)** distal regions. **(C)** Identification and quantification of sites containing known HOMER motifs for regions that increase in accessibility with LPS stimulation as compared with unstimulated, untreated BMDMs (FDR < 0.05 for differential analysis, FDR < 0.10 for motif enrichment). **(D)** Identification, motif clustering, and quantification of sites containing known HOMER motifs for regions that decrease in accessibility with (+)-JQ1 treatment prior to LPS stimulation as compared with LPS stimulation alone (FDR < 0.05 for differential analysis, FDR < 0.10 for motif enrichment). **(E)** Gene tracks of ATAC-seq peaks within 25kb of the transcription start site of *Il12β* for unstimulated (top), LPS + (+)-JQ1 (center) and LPS stimulated (bottom) conditions. Horizontal purple bars above the tracks are representative of peaks that are differentially increased during LPS stimulation compared with unstimulated BMDMs and are differentially decreased with (+)-JQ1 as compared with LPS alone (FDR < 0.05). Red line is representative of putative AP-1 motif identified by HOMER. Numbers to the right represent corresponding *Il12β* transcript levels. FDR adjusted P values for differential analysis determined using DESeq2 and for motifs using HOMER. N = 3 biological replicates.

Distal regulatory elements, such as enhancers, largely contribute to cell-specific gene expression programs defining cell identity (4, 5). Recent studies have suggested that BET protein inhibition alters use of cell-specific super-enhancers (56–58). Therefore, we sought determine the relationship between effects of (+)-JQ1 on chromatin accessibility and altered expression at LPS-responsive genes. Across our ten gene expression classes, we found a similar number of distal accessible regions near member genes on average (+/- 25kb from the TSS; **Supplementary Figures 5A, B**). LPS stimulation overall increased distal chromatin accessibility in all classes, but the magnitude of this increase varied (**Supplementary Figure 5C**). Consistent with our genome-wide analyses, (+)-JQ1 treatment decreased the effect of LPS stimulation on chromatin accessibility changes for all 10 classes, most notably for Classes VI, VII, and IX that contain genes with attenuated expression (**Figures 3A, B**; **Supplementary Figure 4C**). In contrast, a similar analysis of IL-10 treated samples on Class VI, VII, and IX genes showed accessibility at promoter regions were even less altered on average than with (+)-JQ1 treatment (**Supplementary Figure 5D**). However, accessibility at distal regions associated with these classes more closely resembled those of untreated LPS stimulated samples, especially for Class VII, as compared with (+)-JQ1 treatment (**Supplementary Figure 5E**).

Focusing more specifically on regions that significantly changed upon LPS stimulation, we found an enrichment for DARs near genes from Classes VI (n=504) and VII (n=47), but not Class IX (n=66) (hypergeometric test; p = 9.22x10^-16^, p = 7.56x10^-4^, p = 0.24, respectively). As Class VI was the largest and contains key inflammatory genes, we further investigated chromatin accessibility separately in promoter and distal regions. Both sets of regions showed increased chromatin accessibility upon LPS stimulation compared with unstimulated *Il10^-/-^* BMDMs (**Figures 3A, B**). Consistent with our genome-wide observations, (+)-JQ1 treatment showed little effect on promoter regions (**Figure 3A**), but distally we found an overall attenuation of accessibility changes and a reduced number of DARs in response to LPS stimulation (**Figure 3B**). Motif analysis of distal DARs associated with Class VI genes with increased accessibility in untreated LPS stimulated samples (n = 5,323 regions) showed enrichment for motifs of bZIP transcription factors, including many AP-1 family sub-units, ETS transcription factors, including PU.1, and IRF family members (**Figure 3C**). DARs with attenuated accessibility changes in (+)-JQ1 treated samples during LPS stimulation compared with LPS stimulation alone (n = 920 regions) revealed motifs for a subset of AP-1 family members as well as several IRF and RHD (NF-kB p65/p50) factors (**Figure 3D**). This included a putative AP-1 motif located downstream of the of the *Il12β* TSS (**Figure 3E**). DARs that decreased in accessibility during LPS stimulation alone or increased with (+)-JQ1 treatment with LPS were enriched for motifs from ETS family members (**Supplementary Figures 5F, G**).

Overall, these data suggest that (+)-JQ1 treatment restricts LPS-induced chromatin accessibility changes at distal regulatory elements near inflammatory genes. Interestingly, though, our data also suggests that exogenous IL-10 may also attenuate some LPS-induced chromatin changes, but primarily at promoters of LPS-inducible genes.

### (+)-JQ1 Treatment Limits Onset of Experimental Colitis in Germ Free *Il10^-/-^* Mice

**Figure 4:**
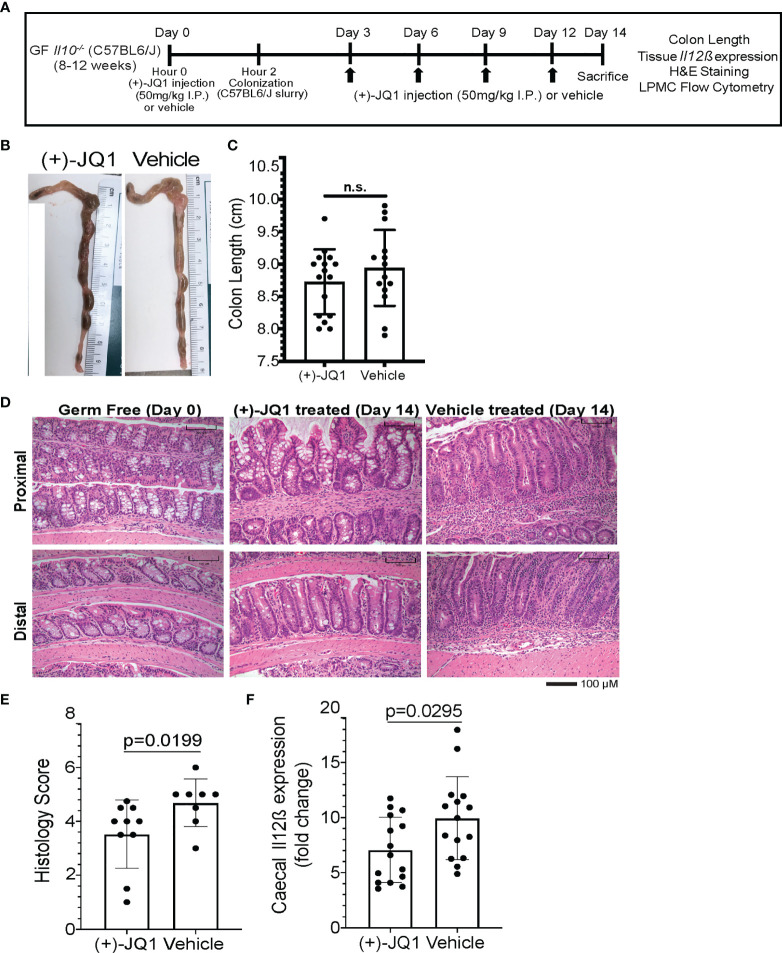
(+)-JQ1 treatment attenuates onset of microbiota-induced colitis in germ free *Il10^-/-^* mice. **(A)** Schematic of experimental workflow (n = 10+ mice/condition and represent at least 3 independent experiments). **(B)** Representative images of colon length and thickness in (+)-JQ1- and vehicle-treated mice. **(C)** Quantification of average colon length (n = 16 (+)-JQ1; n = 15 vehicle). **(D)** Representative images of H&E stained colons for proximal (top) and distal (bottom) portions of the colon in germ free (GF) mice (left), colonized mice treated with (+)-JQ1 harvested on Day 14 (center) and colonized mice treated with vehicle harvested on Day 14 (right). Scale represents 100 µM. **(E)** Quantification of histology scores based on H&E staining (n = 10 (+)-JQ1; n=8 vehicle). **(F)** Quantification of caecal *Il12β* expression by qPCR (n = 15/treatment). Significance values determined using a 2-tailed unpaired, non-parametric student’s t test. N.S. stands for Not Significant.

To determine whether (+)-JQ1 may mitigate colitis onset *in vivo*, we treated adult *Il10^-/-^* mice previously housed under germ free (GF) conditions with (+)-JQ1 (I.P. injections) followed by colonization with slurries made using fecal matter from WT C57BL/6J mice raised in specific pathogen free conditions (**Figure 4A**). Additional (+)-JQ1 treatments were administered on Days 3, 6, 9, and 12 and animals were sacrificed on Day 14 (**Figure 4A**). Treatment with (+)-JQ1 did not result in any significant changes in weight as compared with vehicle treated mice (**Supplementary Figure 6A**).

We isolated lamina propria mononuclear cells (LPMCs) and splenocytes from (+)-JQ1- and vehicle treated animals to identify changes in immune cell population sizes associated with treatment (**Supplementary Figure 6B**). Total CD45^+^ leukocytes constituted ~60% of singlets in LPMC populations and 75% of singlets in splenocyte populations in vehicle treated mice and did significantly change with (+)-JQ1 treatment (**Supplementary Figure 6C**). No significant differences in the population size of CD11b^+^CD11c^-^F4/80^+^ macrophages or total CD3^+^ T cells and CD19^+^ B cells isolated from the intestine or spleen were observed with (+)-JQ1 treatment (**Supplementary Figures 6D, E**). However, a reduction in the CD11b^int^CD11c^-^ population was observed with (+)-JQ1 treatment (**Supplementary Figure 6B**). Based on these observations, we conclude that the dose and frequency of (+)-JQ1 given to these mice does not have any significant effect on bulk immune cell population size within the intestine or other peripheral organs.

Finally, we investigated the effect of (+)-JQ1 treatment on intestinal inflammation in these mice. Overall colon length and morphology remained unchanged with (+)-JQ1 treatment when compared with vehicle treated mice (**Figures 4B, C**). However, histologically, scored using a modified method from Berg et al. that compared colons with uninflamed uncolonized germ-free *Il10^-/-^* mice harvested on Day 0 (**Figure 4D**), (+)-JQ1 treated mice had statistically significantly lower scores compared with vehicle treated mice (**Figure 4E**). Additionally, we quantified caecal *Il12β* mRNA expression levels and found that (+)-JQ1 treatment also significantly decreased relative expression as compared with vehicle treated mice (**Figure 4F**), which correlates with our histological observations. Taken together, these data demonstrate that treatment with (+)-JQ1 attenuates severity of colitis onset in *Il10^-/-^* mice two weeks post colonization.

## Discussion

The enhancer landscape is central to determining the gene expression program and overall identity of a cell (4, 5). Both the microenvironment and external stimuli influence macrophage enhancer utilization (4, 5, 8–11). Previously, we showed that putative enhancers regions are differentially accessible in a genotype- and stimulation-dependent manner in *Il10^-/-^* macrophages (9). In this study, we determined that BET protein inhibition using (+)-JQ1 is sufficient to attenuate LPS-induced chromatin remodeling at putative enhancers containing AP-1 and IRF motifs distal to the TSS of key inflammatory genes whose expression are induced by LPS and show concomitant attenuation with (+)-JQ1. BET proteins, particularly BRD4, increase in binding to chromatin in macrophages following LPS stimulation (30, 56). Furthermore, BRD4 is important for the formation of LPS-induced super-enhancers in close proximity to inflammatory genes such as *Tnf* and *Nfkbia* (56). Additional evidence has also suggested a potential role for BRD2 in controlling transcriptional activation by binding the chromatin insulator CTCF and the cohesion to promote cis-regulatory enhancer assembly (59). We propose that treatment with (+)-JQ1 inhibits BET protein-mediated nucleosome eviction thus preventing LPS-induced increases in chromatin accessibility at putative enhancers driving inflammatory gene expression (36). Our studies are limited by the absence of ChIP-seq datasets and functional assays that confirm the regions identified in this study are enhancers. Future studies should annotate these regions for H3K4me1 and H3K27ac marks as well as utilize luciferase-reporter assays and electrophoretic mobility shift assays to confirm enhancer activity and identify the exact transcription factors associated with promoting regulatory activity.

BET protein inhibition results in decreased posttranslational modification of transcription factors, including NF-kB, AP-1 and STAT5, thus limiting expression of genes that rely on these proteins (60–62). Furthermore, BET protein recognition of K310ac on RelA is also central to NF-kB transcriptional activation (63). While we show (+)-JQ1 having a limited effect on attenuating LPS induced accessibility at putative p65 and p50 motifs, its impact on post translational modification of these subunits suggests different mechanisms through which BET protein inhibition attenuates inflammation. We also show loss of BET protein binding following LPS stimulation limits production of other transcription factors, such as IRF family members, which are responsible for additional chromatin modification to activate other inflammatory genes (64, 65).

To our knowledge, there are no studies that have determined if any of the chromatin-based effects of (+)-JQ1 are permanent following cessation in culture. Gibbons et al. recently demonstrated that (+)-JQ1 inhibits expression of IFN-γ in T_H_1 cells, but this blockade only resulted from the absence of BET protein binding at the promoter, not remodeling of chromatin associated with known enhancers for IFN-γ (34). Consequently, removal of (+)-JQ1 from culture resulted in the restoration of IFN-γ expression. In contrast, other studies have demonstrated blockade of chromatin modifiers is sufficient to have long-term consequences (66). Future studies are necessary to determine if (+)-JQ1-mediated inhibition of LPS-induced increased at putative AP-1 and IRF motifs are permanent following removal of (+)-JQ1 from culture and should include re-challenge with LPS to assess macrophage tolerization (67).

Previous publications have focused on pairwise differential gene expression analyses in macrophages between LPS and LPS + BET inhibitor or BET knock-out conditions, and consistently highlight the relative down regulation of inflammatory genes (30, 32, 37, 56). However, gene expression in unstimulated macrophages are seldom included in these studies despite the fact many inflammation-related surface receptors are expressed and central to macrophage functioning at baseline. Our study utilized an objective approach that presents a genome-wide annotation of LPS-stimulated gene expression changes and how (+)-JQ1 modulates this response relative to the expression observed in an unstimulated macrophage. While our findings regarding the attenuation of inflammatory genes with BET inhibition is consistent with prior studies, we demonstrate that the majority of genes induced by LPS stimulation still show increased expression with (+)-JQ1 treatment compared to unstimulated macrophages (Class VI). These data indicate that BET inhibition by (+)-JQ1 does not completely ablate the ability of macrophages to respond to a bacterial stimulus. However, our analytical approach identified additional gene expression dynamics associated with (+)-JQ1 treatment where a subset of LPS-induced genes had expression levels below that of unstimulated macrophages (Class VII) and another subset had expression levels that were comparable to unstimulated macrophages (Class IX). These include *Fcgr1*, *Fcgr2b*, *Fcgr4* (Class VII), *Tlr1*, *Tlr6*, and *Cd80* (Class IX), which are involved in antibody-mediated phagocytosis, bacterial signaling, and co-stimulation of T cells. Genomic data alone is not sufficient to determine the functions retained by macrophages in the presence of (+)-JQ1 and little data regarding BET inhibitor-mediated modulation of macrophage functioning exists (68). Therefore, it is necessary for future studies to determine if macrophages retain functional their phagocytic and functional capabilities upon and following BET inhibition.

We previously proposed that IL-10 signaling may promote the early establishment of accessible regulatory elements in the developing macrophage (9). Our findings here illustrate a novel role for IL-10 in facilitating chromatin remodeling, but it remains unclear if IL-10 interacts with chromatin directly or if these changes are prompted by downstream effectors in the IL-10 pathway. Co-culture of *Il10^-/-^* BMDMs with exogenous IL-10 is sufficient to alter the LPS-induced chromatin landscape but is distinct from the resulting chromatin landscape associated with BET inhibition. Chromatin profiles of samples treated with IL-10 or (+)-JQ1 prior to LPS stimulation more closely resemble unstimulated samples than untreated LPS stimulated samples, but these treatments generate distinct chromatin profiles. Interestingly, IL-10 was more potent in attenuating promoter accessibility changes, whereas (+)-JQ1 had a larger effect on distal regions. This observation, coupled with similar upregulation of LPS-induced genes as untreated LPS stimulated samples, suggests that IL-10 treatment is not sufficient to prevent chromatin changes that drive the upregulation of inflammatory genes. In contrast, (+)-JQ1 treatment predominantly resulted in the attenuation of LPS-induced gene expression, including genes involved in anti-inflammatory pathways promoted by IL-10, including *Stat3* and *Jak2* (69). Previously, it was shown the IL-10 serum levels were significantly lower in an LPS-induced endotoxic shock model when mice were treated with (+)-JQ1 (31). More recent findings have demonstrated that IL-10 producing B regulatory cells rely on BRD4 to promote expression of IL-10 (70). Together, these data, along with our own results, suggest that BET inhibition results in the repression of key anti-inflammatory pathways, in addition to inflammatory pathways. Future experiments should further interrogate the effects of (+)-JQ1 on macrophage LPS-induced gene expression with intact IL-10 signaling to determine the potential short and long-term consequences that may result due to the unintended inhibition of a key anti-inflammatory pathway.

The ability of BET protein inhibitors to attenuate inflammation by modulating the function of macrophages in colitis is largely unexplored. Although Cheung et al. demonstrated that treatment with BET inhibitors limited T_H_1 and T_H_17 differentiation and prevented adoptive T-cell transfer induced colitis, Weinerroither et al. revealed that the use of BET inhibitors at the onset of DSS-induced colitis exacerbated the phenotype as compared with DSS administration alone (37, 68). It is unclear whether changes in macrophage phenotype impacts T cell expansion and differentiation. Similarly, the DSS-model of colitis is an acute injury model that damages the intestinal epithelium, suggesting that (+)-JQ1 may prevent the expansion and differentiation of intestinal stem cells, resulting in worse tissue injury (71, 72). Our study presents the first evaluation of BET inhibitors as a method of mitigating onset of colitis severity in a genotype-driven, microbiota-dependent mouse model. There are limitations to our study. While our overall phenotype shows that (+)-JQ1 treatment results in modest reduction in colitis severity as compared with vehicle-treated mice, further studies are needed to identify the optimal dosing strategy that maximizes attenuation of inflammation without causing harm to the intestinal epithelium (71). The lack of differences in bulk T and B cells suggests (+)-JQ1 treatment may alter cell phenotype, as was previously observed in the adoptive T-cell transfer-induced colitis model when treated with a BET inhibitor (37). Understanding the effects of (+)-JQ1 on the genome wide regulatory landscape macrophages within the intestine is largely hindered by low yield of live macrophages an essential requirement for high quality/quantity RNA for sequencing studies. While our study focused on genomic changes associated with BMDMs, the decreased caecal expression of *Il12β*, a gene confirmed to be attenuated with (+)-JQ1 in BMDMs, suggests a similar attenuation of innate immune responses within the intestine.

In summary, our study provides evidence that BET inhibitors have the ability to overcome dysregulated inflammatory signaling in *Il10^-/-^* macrophages and accomplishes this through prevention of bacterial stimulation dependent chromatin remodeling. Due to the heterogeneous nature of both CD pathogenesis and presentation, it will be necessary to isolate LP MΦs from CD patients to identify underlying chromatin aberrancies driving inflammation and determine if our results can be recapitulated in *ex vivo* co-culture systems prior to the pursuit of clinical trials.

## Data Availability Statement

The datasets presented in this study can be found in online repositories. The names of the repository/repositories and accession number(s) can be found below:

https://www.ncbi.nlm.nih.gov/geo/query/acc.cgi?acc=GSE183563

https://www.ncbi.nlm.nih.gov/geo/query/acc.cgi?acc=GSE183564

https://www.ncbi.nlm.nih.gov/geo/query/acc.cgi?acc=GSE183565.

## Ethics Statement

The animal study was reviewed and approved by University of North Carolina at Chapel Hill IACUC (19-108.0).

## Author Contributions

MHO, TSF, and SZS conceptualized the idea for the study and wrote the manuscript. MHO, RBS, TSF, and SZS designed the experiments. MHO, AB, ZJL, MRS, ECS, JH, and OKT performed the experiments. MHO, TF, MMKN, BPK, and JMS analyzed the sequencing data. WJ and JCA scored the histology. SZS and TSF supervised the project. SZS funded the project. All authors contributed to the article and approved the submitted version.

## Funding

This work was funded in part through Helmsley Charitable Trust (SHARE Project 2), NIDDK P01DK094779, NIDDK 1R01DK104828, NIDDK P30-DK034987, NIDDK 1R01DK124617, NIH Ruth L. Kirschstein National Research Service Award Individual Predoctoral Fellowship (1F31DK122704), NIH T32 Genetics NIGMS Training Grant (T32-GM007092-43), NIH T32 Translational Medicine Training Grant (T32-GM122741), Crohn’s and Colitis Foundation Student Research Fellowship Award (528437), and Gnotobiotic Animal Facility.

## Conflict of Interest

The authors declare that the research was conducted in the absence of any commercial or financial relationships that could be construed as a potential conflict of interest.

## Publisher’s Note

All claims expressed in this article are solely those of the authors and do not necessarily represent those of their affiliated organizations, or those of the publisher, the editors and the reviewers. Any product that may be evaluated in this article, or claim that may be made by its manufacturer, is not guaranteed or endorsed by the publisher.
