# Supplementary material for: BET Protein Inhibition Regulates Macrophage Chromatin Accessibility and Microbiota-Dependent Colitis
Source: Front Immunol. 2022 Mar 24;13:856966. doi: 10.3389/fimmu.2022.856966 (PMC8988134; doi:10.3389/fimmu.2022.856966)
Supplement: Supplementary file 1 [file DataSheet_1.docx]

**Supplementary Figures**

**
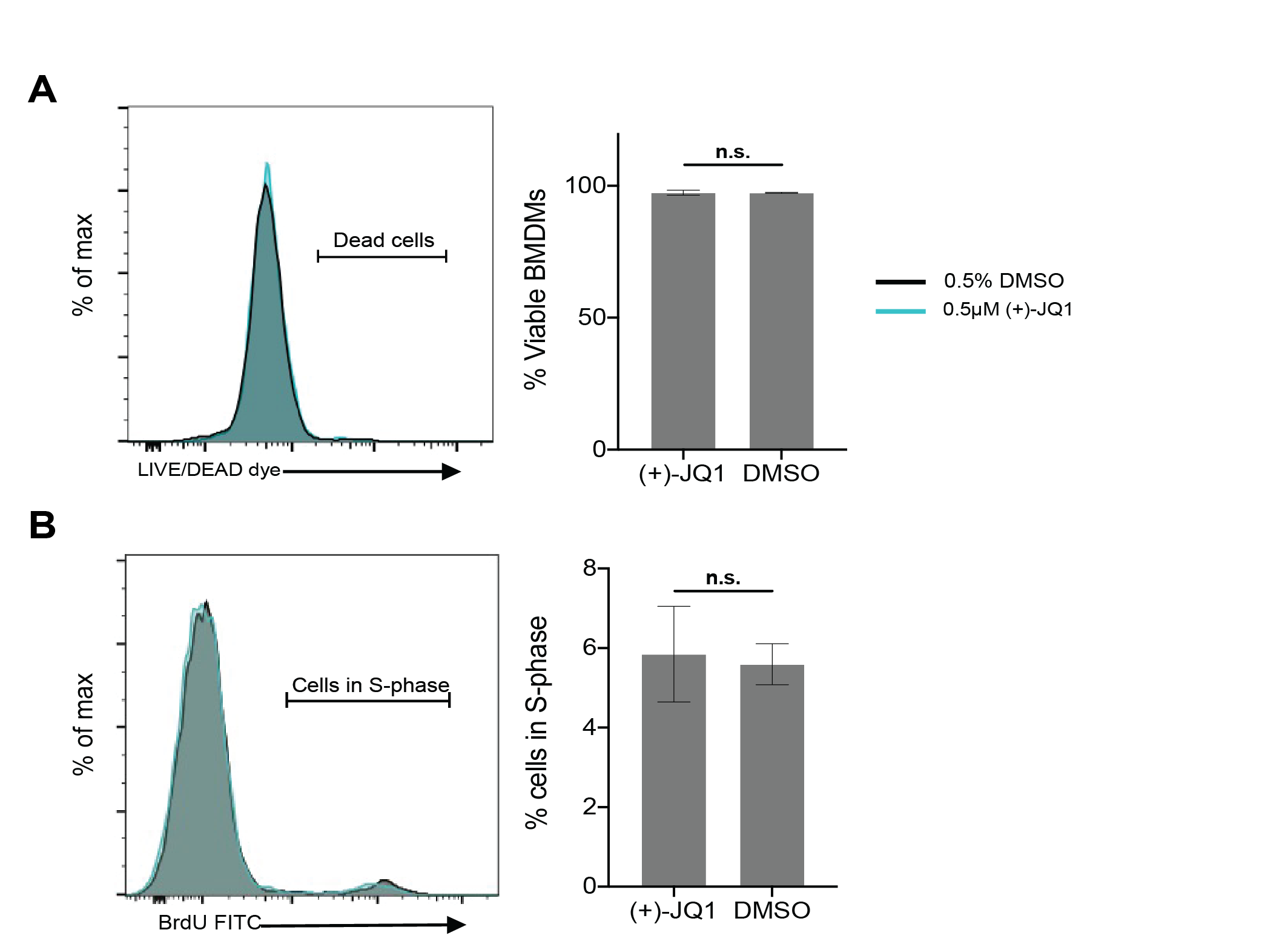
**

**Supplementary Figure 1­.** **Pro-longed (+)-JQ1 treatment does not alter viability or cell cycle progression in BMDMs.** (A) Evaluation and quantification of (A) LIVE/DEAD dye or (B) BrdU FITC incorporation in BMDMs treated with (+)-JQ1 or 0.5% DMSO. Statistics generated using paired, 2-tailed student’s t-test. N=4 biological replicates for viability analysis and N=3 biological replicates for cell cycle analysis.

**
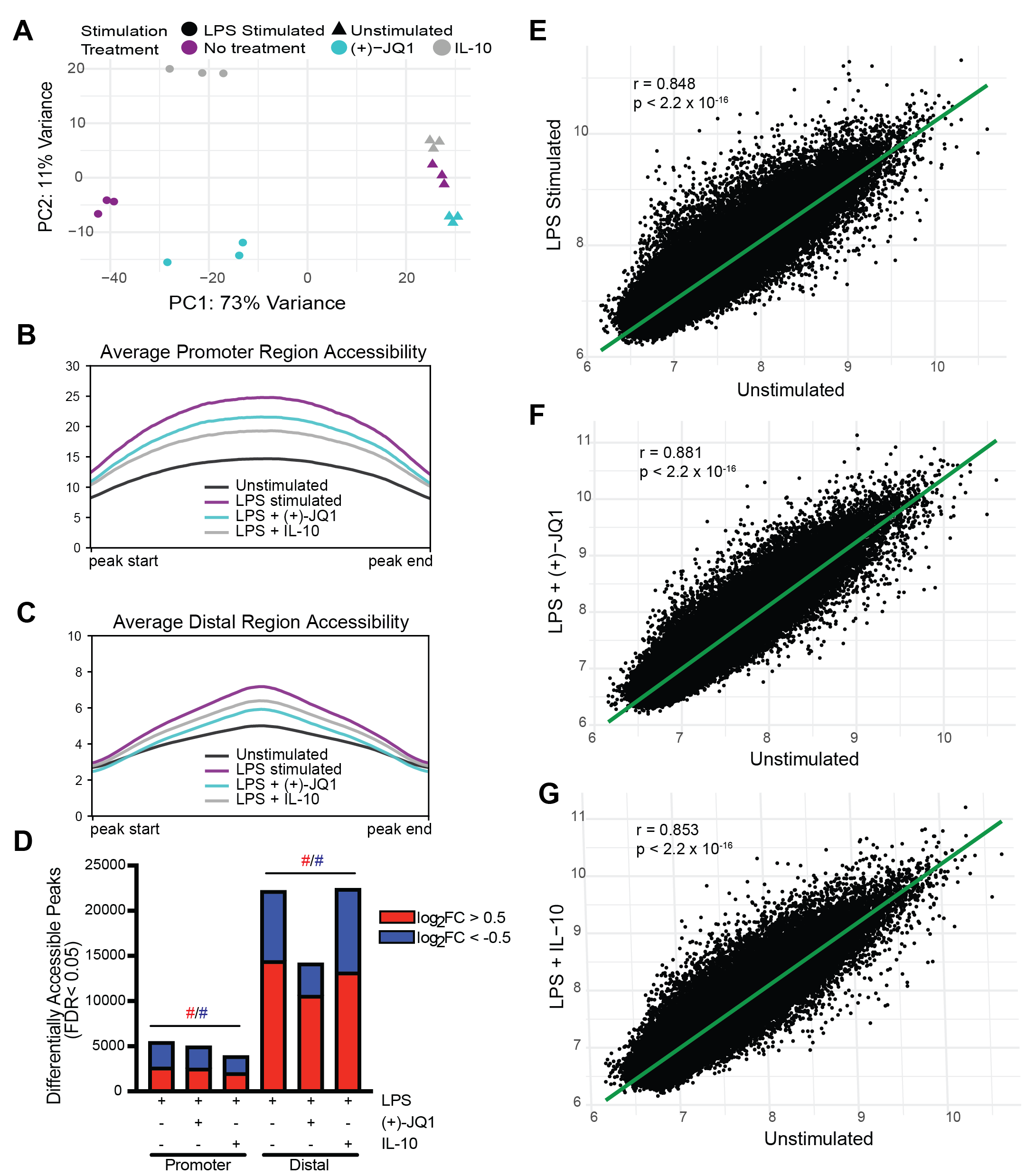
Supplementary Figure 2.** **Chromatin remodeling with (+)-JQ1 treatment is distinct from exogenous IL-10.** (A) Principle component analysis (PCA) of chromatin accessibility profiles for samples that remained unstimulated (triangles) or were stimulated with LPS (circles) in the absence of treatment (purple) or presence of (+)-JQ1 (aqua) or IL-10 (gray). Average chromatin accessibility signal profiles for (B) promoter regions (-1000/+100 bases) and (C) distal regions for unstimulated (black), LPS stimulated (purple), LPS + (+)-JQ1 (aqua) and LPS + IL-10 (gray) samples. (D) Identification of regions that differentially increase (red) or decrease (blue) in accessibility during LPS stimulation with or without (+)-JQ1 or IL-10 as compared with unstimulated *Il10^-/-^* BMDMs at promoter (left) and distal (right) regions (FDR < 0.05). Red and blue hashtags indicate difference in the number of regions with increased (red) or decreased (blue) accessibility with IL-10 is statistically significant using a chi-square test. Spearman rank correlations using VST transformed counts correlating unstimulated counts with (E) LPS stimulation (rho = 0.848), (F) LPS + (+)-JQ1 (rho = 0.881), and (G) LPS + IL-10 (rho = 0.853) conditions. FDR adjusted P values determined using DESeq2. Spearman rank correlations determined by RStudio. N=3 biological replicates.

**
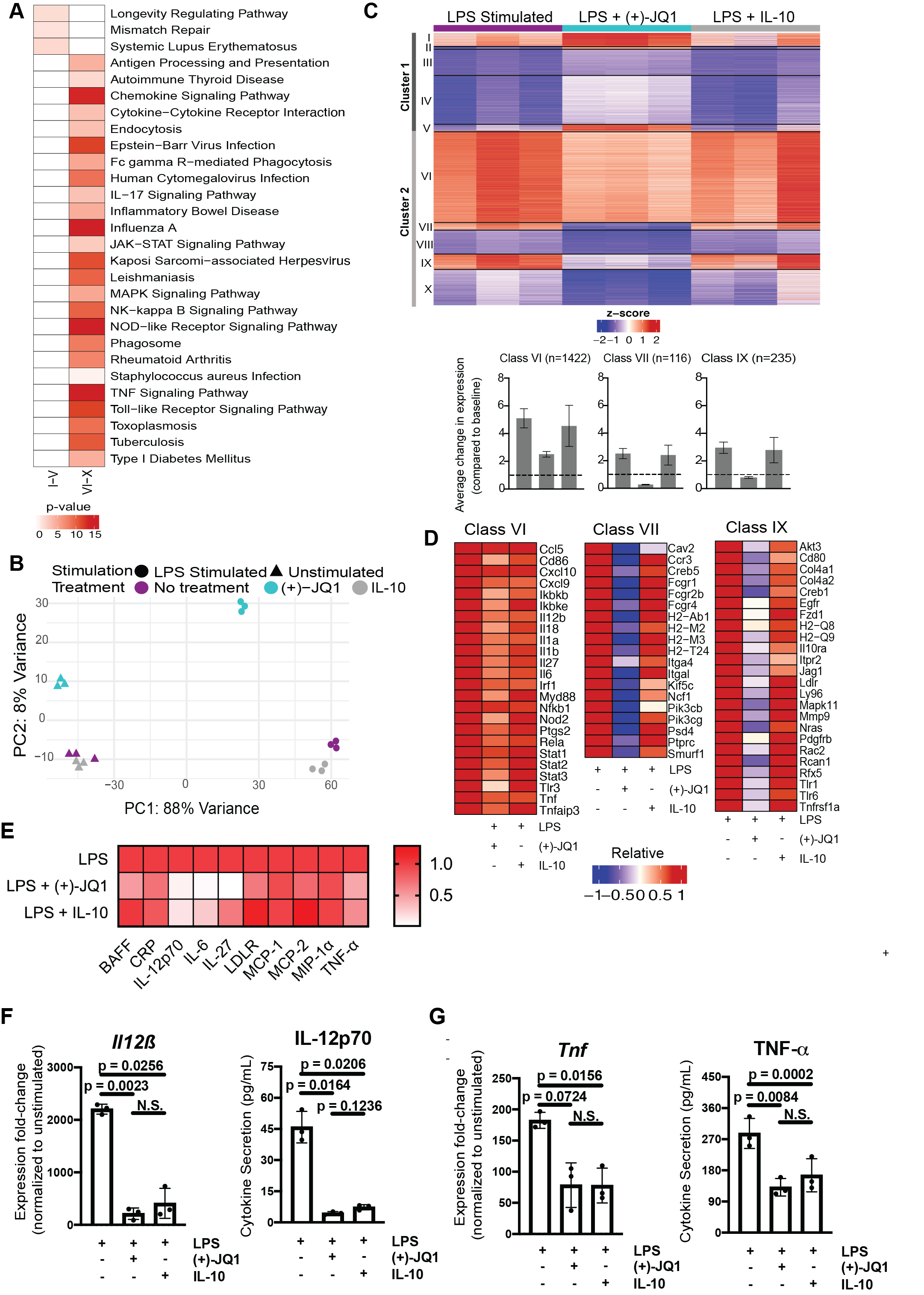
Supplementary Figure 3. Exogenous IL-10 has limited impact on LPS-induced inflammatory gene expression as compared with (+)-JQ1.** (A) KEGG pathway analysis for all genes in cluster 1 (Classes I – V) and cluster 2 (Classes VI – X) (B) Principle component analysis (PCA) of gene expression profiles for samples that remained unstimulated (triangles) or were stimulated with LPS (circles) in the absence of treatment (purple) presence of (+)-JQ1 (aqua) or IL-10 (gray). (C) Representative heatmap of calculated z-scores based on log_2_ fold-change of individual replicates for the LPS, LPS + (+)-JQ1, and LPS + IL-10 conditions normalized to the average of unstimulated, untreated *Il10^-/-^* BMDMs. Genes were identified and divided into 2 major clusters and sub-divided into 5 classes through differential analyses outlined in Supplementary Table 1. Bar graphs represent average change in expression for LPS, LPS (+)-JQ1, and LPS + IL-10 conditions normalized to baseline for Classes. VI, VII, and IX. (D) Heatmaps of representative genes from Classes VI, VII, and IX that correspond to genes induced by LPS that are attenuated with (+)-JQ1 and compare to addition of IL-10. (E) Heatmaps of representative inflammatory cytokines induced by LPS that are attenuated with (+)-JQ1 and compared to the addition of IL-10. Gene expression (qRT-PCR; left panels) and cytokine secretion (Luminex; right panels) analyses for (F) *Il12β* and IL-12p70 and (G) *Tnf* and TNF-α. Significance values determined using a one-way ANOVA followed by Tukey’s multiple comparisons test. N=3 biological replicates.


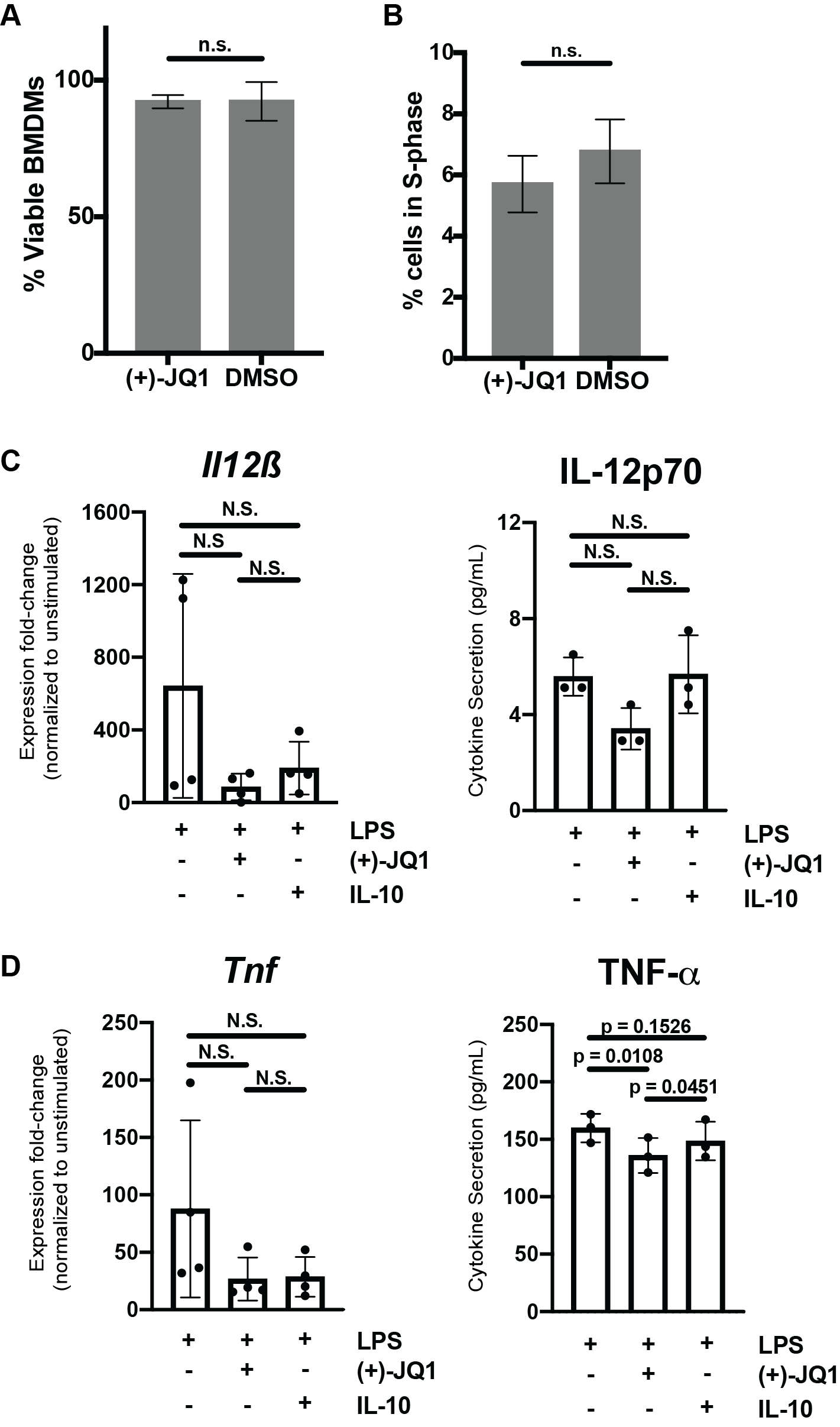


**Supplementary Figure 4. (+)-JQ1 and IL-10 pre-treatment have less pronounced effects on LPS-induced gene expression in WT cells.** (A) Evaluation and quantification of (A) LIVE/DEAD dye or (B) BrdU FITC incorporation in BMDMs treated with (+)-JQ1 or 0.5% DMSO. Gene expression (qRT-PCR; left panels) and cytokine secretion (Luminex; right panels) analyses for (C) *Il12β* and IL-12p70 and (D) *Tnf* and TNF-α. N=4 biological replicates for viability analysis and N=3 for cell cycle analysis. Statistics generated using paired, 2-tailed student’s t-test for flow cytometry experiments. Significance values determined using a one-way ANOVA followed by Tukey’s multiple comparisons test for qRT-PCR and Luminex. N=4 biological replicates for viability analysis and N=3 biological replicates for cell cycle analyses and Luminex analyses.


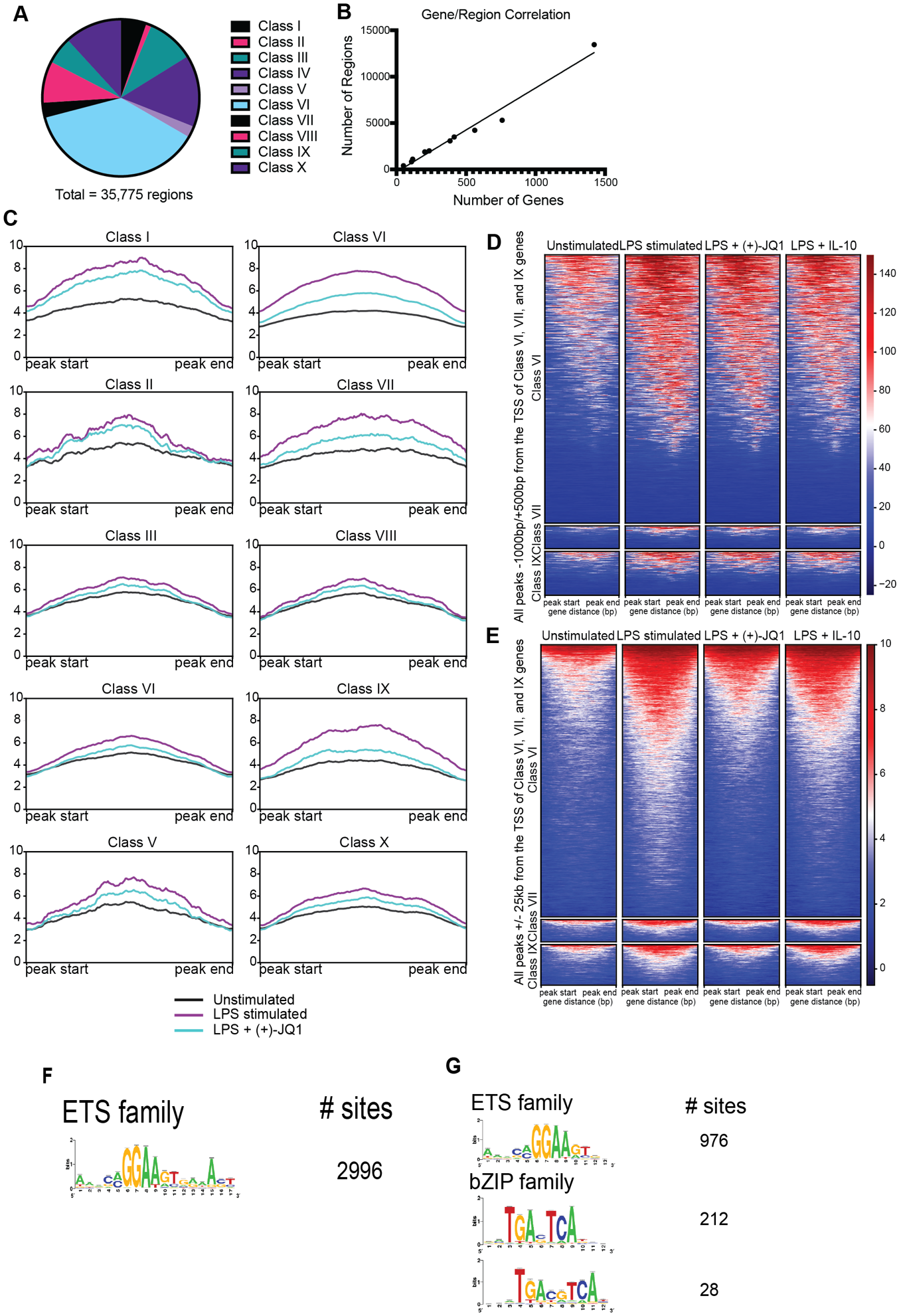


­**Supplementary Figure 5. Lack of LPS-induced chromatin remodeling at distal regions associated with Class VI is unique to (+)-JQ1 treatment.** (A) Pie chart representing the number of distal peaks associated with each gene class using BEDtools intersect. (B) Correlation of the number of DEGs associated with each gene class and the number of peaks identified (R^2^=0.9762, p<0.0001). (C) Average signal plots for all distal regions associated with Classes I – X. Heatmaps of accessible chromatin during unstimulated (left), LPS stimulated (center), LPS + (+)-JQ1, or LPS + IL-10 conditions for Classes VI (top), VII (center), and IX (bottom) for ChIPSeeker identified (D) promoter regions and (E) distal regions. Identification, motif clustering, and quantification of sites containing known HOMER motifs for regions that (F) decrease in accessibility with LPS stimulation as compared with unstimulated BMDMs or (G) increase with (+)-JQ1 treatment prior to LPS stimulation as compared with LPS stimulation alone. FDR < 0.05 for differential analysis; FDR < 0.10 for motif enrichment. FDR adjusted P values for differential analysis determined using DESeq2 and for motifs using HOMER. N=3 biological replicates.


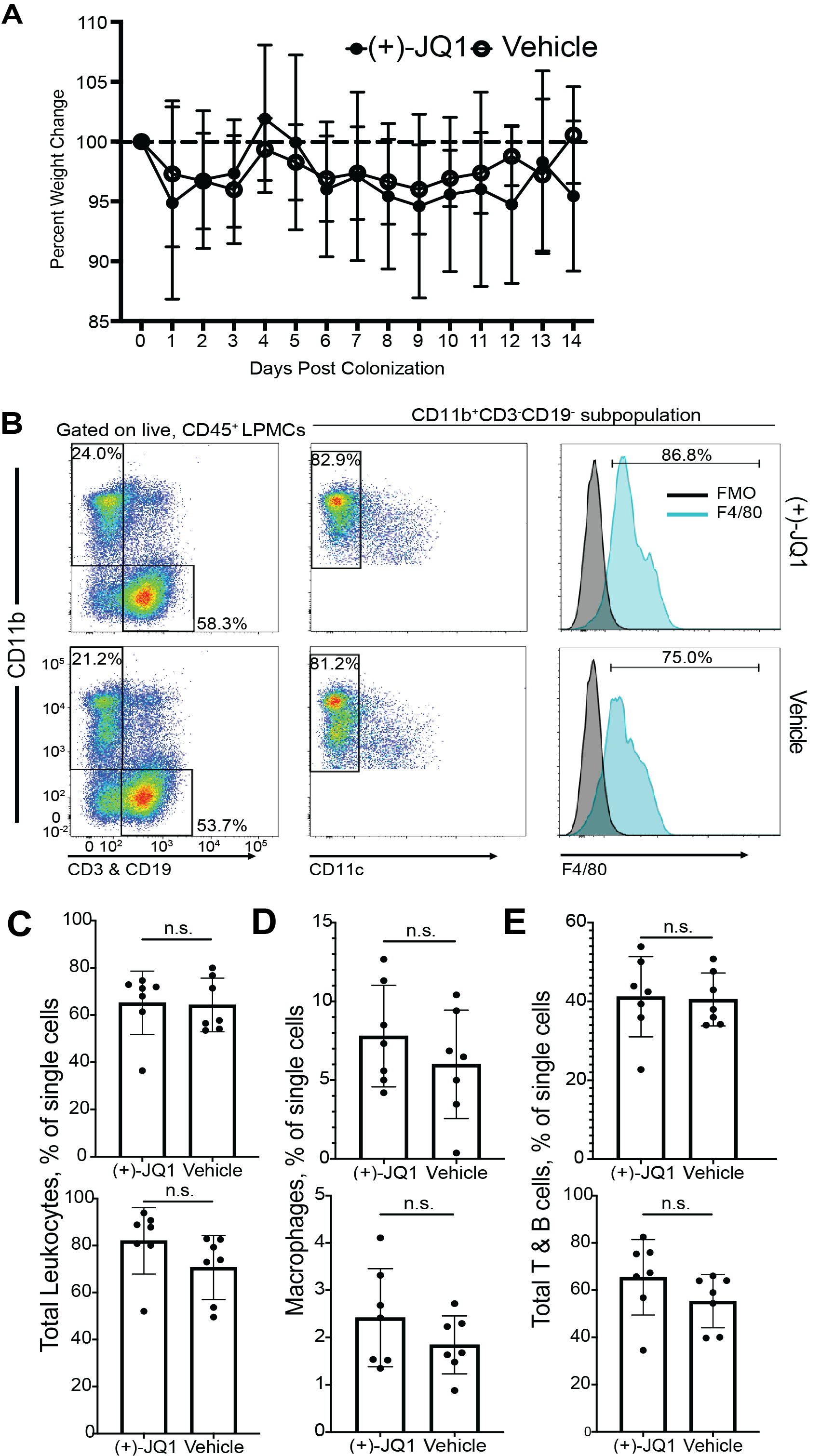


­**Supplementary Figure 6. (+)-JQ1 treatment does not have a significant effect on bulk immune cell population size.** (A) Daily percent change associated with treatment using (+)-JQ1 or vehicle control (n = 16/treatment). (B) Representative gating strategy for CD11b^+^CD11c^-^F4/80^+^ macrophage and CD3^+^/CD19^+^ lymphocyte populations bulk lamina propria mononuclear cells (LPMCs) isolated from murine colons. Quantification of colonic (top) and splenic (bottom) (C) total CD45^+^ leukocytes (D) CD11b^+^CD11c^-^F4/80^+^ macrophages and (E) CD3^+^ & CD19^+^ lymphocyte populations normalized to the number of single cells acquired by flow cytometry (n = 7/treatment). Statistics generated using two-tailed, unpaired, student’s t test.

**Supplementary Tables**

Supplementary Table 1. LRT and Wald (pair-wise) test parameters used to identify 10 gene expression classes with (+)-JQ1 treatment and LPS stimulation.

| Class | Log_2_FC Direction (LRT) | P_adj_ (unstimulated vs LPS stimulated) | Log_2_FC (unstimulated vs LPS stimulated) | P_adj_ (unstimulated vs LPS + (+)-JQ1) | Log_2_FC (unstimulated vs LPS + (+)-JQ1) | Number of Genes |
| --- | --- | --- | --- | --- | --- | --- |
| I | Log_2_FC>0 | P_adj_<0.05 | Log_2_FC>0 | P_adj_<0.05 | Log_2_FC>1 | 203 |
| II | Log_2_FC>0 | P_adj_<0.05 | Log_2_FC<0 | P_adj_<0.05 | Log_2_FC>1 | 50 |
| III | Log_2_FC>0 | P_adj_<0.05 | Log_2_FC<0 | P_adj_<0.05 | Log_2_FC<-1 | 415 |
| IV | Log_2_FC>0 | P_adj_<0.05 | Log_2_FC<0 | P_adj_>0.05 | NA | 760 |
| V | Log_2_FC>0 | P_adj_>0.05 | NA | P_adj_<0.05 | Log_2_FC>1 | 108 |
| VI | Log_2_FC<0 | P_adj_<0.05 | Log_2_FC>1 | P_adj_<0.05 | Log_2_FC>0 | 1422 |
| VII | Log_2_FC<0 | P_adj_<0.05 | Log_2_FC>1 | P_adj_<0.05 | Log_2_FC<0 | 116 |
| VIII | Log_2_FC<0 | P_adj_<0.05 | Log_2_FC<-1 | P_adj_<0.05 | Log_2_FC<0 | 285 |
| IX | Log_2_FC<0 | P_adj_<0.05 | Log_2_FC>1 | P_adj_>0.05 | NA | 235 |
| X | Log_2_FC<0 | P_adj_>0.05 | NA | P_adj_<0.05 | Log_2_FC<0 | 563 |
